# Impact of a 20-Week Resistance Training Program on the Force–Velocity Profile in Novice Lifters Using Isokinetic Two-Point Testing

**DOI:** 10.3390/jfmk9040222

**Published:** 2024-11-05

**Authors:** Joffrey Drigny, Nicolas Pamart, Hélène Azambourg, Marion Remilly, Emmanuel Reboursière, Antoine Gauthier, Amir Hodzic

**Affiliations:** 1 Department of Sports Medicine and Physical Medicine and Rehabilitation, CHU de Caen Normandie, Normandie Univ, UFR Santé, GIP Cyceron, INSERM Comete, 14000 Caen, France; 2 Inter-University Laboratory of Human Movement Science (LIBM EA7424), University of Lyon, University Jean Monnet, 42100 Saint-Etienne, France; 3 Department of Sports Medicine, Normandie Univ, UFR Santé, UNICAEN, CHU de Caen Normandie, 14000 Caen, France; 4 Normandie Univ, UFR Santé, GIP Cyceron, INSERM Comete, 14000 Caen, France; 5 Department of Cardiology and Sports Medicine, Normandie Univ, UFR Santé, UNICAEN, CHU de Caen Normandie, INSERM Comete, GIP Cyceron, 14000 Caen, France

**Keywords:** exercise physiology, isokinetic, muscle function, physical performance, strength training

## Abstract

**Objectives**: This study aimed to assess the impact of a 20-week resistance training program on force–velocity (F-V) parameters using an isokinetic two-point method and comparing one-repetition maximum (1-RM) methods in novice lifters. **Methods**: Previously untrained individuals completed a supervised, three-session weekly resistance training program involving concentric, eccentric, and isometric phases, repeated every 2 to 4 weeks. Isokinetic dynamometry measured the strength of elbow flexors/extensors at 60°/s and 150°/s, and knee flexors/extensors at 60°/s and 240°/s at Baseline, 3 months, and 5 months. F-V parameters, including maximal theoretical force (F0) and the F-V slope, were calculated. Participants also performed 1-RM tests for the upper and lower limbs. Repeated measures ANOVA with effect size (η^2^ > 0.14 as large) was used to analyze changes in F-V parameters and repeated measures correlation was used to test their association with 1-RM outcomes. **Results**: Eighteen male participants (22.0 ± 3.4 years) were analyzed. F0 significantly increased for all muscle groups (η^2^ = 0.423 to 0.883) except elbow flexors. F-V slope significantly decreased (steeper) for knee extensors and flexors (η^2^ = 0.348 to 0.695). Knee extensors showed greater F0 gains and steeper F-V slopes than flexors (η^2^ = 0.398 to 0.686). F0 gains were associated with 1-RM changes (r = 0.38 to 0.83), while F-V slope changes correlated only with lower limb 1-RM (r = −0.37 to −0.68). **Conclusions**: The 20-week resistance training program significantly increased F0 and shifted the F-V profile towards a more “force-oriented” state in knee muscles. These changes correlated with improved 1-RM performance. Future studies should include longer follow-ups and control groups.

## 1. Introduction

Since Hill’s first description of muscle contraction dynamics, the force–velocity (F-V) relationship has gained popularity in the context of evaluating muscle mechanics and athletic movement [1]. The F-V relationship explains that as a skeletal muscle shortens more slowly, it can generate a greater force during contraction, and vice versa [2]. Understanding the F-V relationship is crucial for addressing specific athletic demands across different sports and, therefore, optimizing strength and power training to enhance performance [3]. Additionally, the F-V properties have been shown to differ between individuals with varying levels of physical fitness, distinguishing non-active or sedentary individuals from those who are active or strength-trained [4,5]. Thus, assessing the F-V characteristics of athletes has been developed for identifying key strength and power profiles essential for optimizing athletic performance and implementing specific strategies to improve overall athletic capabilities and minimize the likelihood of performance-limiting factors [6,7].

Studies have examined the effects of specific training programs on the force and velocity characteristics, with a significant focus on resistance training [6,8]. Resistance training is a structured and progressive exercise modality that applies resistance or load to the musculoskeletal system, stimulating adaptations in muscle strength, hypertrophy, and power. Resistance training induces velocity-specific training adaptations with greater gains in strength in the high force–low velocity domains compared to higher-velocity movements. Thus, it has been suggested that F-V parameters could serve as a basis for individualized resistance-training programs for athletes [9].

The F-V relationship in muscle physiology, as initially described by A.V. Hill in isolated skeletal muscle fibers, follows a hyperbolic curve [10]. However, when examining F-V profiles in human movements and functional tasks, the relationship tends to exhibit a more linear pattern with a negative slope and specific F-V relationship parameters (force intercept [F0], velocity intercept [v0], and F-V slope) [11,12]. This linear model has given the opportunity to define an F-V profile when measuring by capturing data at distinct loads or resistances and corresponding velocities during exercise tasks [13]. Isokinetic dynamometry is considered the gold standard for testing maximal strength for sports and rehabilitation purposes, as it allows for the assessment of maximal strength at a predefined constant velocity. Isokinetic testing is highly suitable for assessing muscle performance in non-athletes and novices, as it is reliable even in inexperienced individuals [14], requires no complex motor skills for assessing muscle function unlike multi-joint functional movements, and offers detailed insights into muscle strength and power at various velocities [15]. Thus, a two-point isokinetic model employing two measures covering a broad range of angular velocities has been demonstrated for assessing the F-V profile of single-joint movements [16,17]. Indeed, the two-point method for assessing force–velocity is easier and fatigue-free, further enhancing its suitability for non-athletes [18]. This two-point method is a valid and sensitive procedure for determining the maximal capacities of both upper and lower limb muscles, especially in the force domain [19,20]. However, the sensitivity to change in this measure has not been previously examined, especially after a resistance training program.

Maintaining muscle balance is crucial for efficient movement patterns, joint stability, and injury prevention in sports and daily activities [21]. It has been suggested that the isokinetic two-point method could differentiate between the mechanical properties of agonistic muscles [17]. Indeed, agonistic muscles are heterogeneous with respect to fiber type and metabolic properties and may experience distinct adaptations to the same stimulus. Therefore, the F-V characteristics may vary between agonist and antagonist muscles based on their specific functions and contraction types in human activities, but the relative adaptations of agonist–antagonist balance to resistance training remain unclear.

Besides isokinetic testing, the one-maximum repetition (1-RM) method, defined as the maximal weight that can be lifted once, is considered the gold standard for reliably assessing muscle strength in non-laboratory situations. If both isokinetic testing and the 1-RM method are useful for evaluating changes in muscle strength after resistance training programs, they might not be equivalent as the magnitude of change shows large variations and can even be conflicting [22]. Thus, authors have tested the association between the maximal strength measured during the 1-RM tests and the maximal force calculated using the F-V profiling with some uncertainty [23,24]. Indeed, it is unclear if changes in the F-V profile and 1-RM tests are consistent after a resistance training program.

The main objective of this study was to test the impact of a 20-week resistance training program on the force–velocity (F-V) profile of upper and lower limb strength in novice lifters, with an analysis focused on agonistic muscles. The second objective was to test the association between the F-V parameters and the 1-RM performance over time.

We hypothesized that resistance training would result in a significant change in the F-V profile with a greater gain in the high force–low velocity domains of the F-V relationship. In addition, we hypothesized that the changes in F-V parameters would be correlated with the changes in the performances over time.

## 2. Materials and Methods

### 2.1. Ethical Considerations

This was a longitudinal cohort study among healthy novice lifters. All subjects provided written informed consent in accordance with the Declaration of Helsinki. This study was approved by an independent ethics committee (EUDRACT: 2019-A01235-52). A compensatory allowance of EUR 150 (USD 158) was provided to all participants who completed the entire study, intended to cover transportation costs and as a token of appreciation for their involvement. This study was registered with Clinical Trials (NCT04187170).

### 2.2. Population

Participants were recruited from the university community. Inclusion criteria were untrained adults aged 18–40 years. Eligible participants were free from known cardiovascular, liver, renal, respiratory, and metabolic diseases, non-smokers, non-hypertensive, non-diabetic, and had a body mass index <35 kg·m^−2^. Before being included in this study, candidates underwent a comprehensive medical evaluation, which included an electrocardiogram, cardiopulmonary exercise testing, and an echocardiogram. These assessments ensured there were no contraindications to resistance training and evaluated exercise capacity. Individuals showing abnormalities in initial examinations, as well as those with cardiopulmonary capacities at or above 120% of the predicted VO_2_ max—used as a threshold for athletic conditioning—were excluded. Twenty-seven male volunteers, aged 18 to 40 years, were included in this study. None of the participants practiced more than 75 min of sustained physical training (endurance or strength training) per week in the three years leading up to this study. No participants declared the use of drugs or doping products. There was neither randomization nor a control group in this study.

### 2.3. Design

This study was part of a broader investigation aimed at examining cardiac morphological and neuromuscular adaptations in a group of healthy, previously untrained young men undergoing a 20-week longitudinal high-intensity resistance-training program [25]. For the present study, all participants underwent a clinical examination at Baseline (prior to the resistance program) that included body composition and isokinetic dynamometry at the elbow and knee joints as described below. These assessments were repeated at the 12-week (e.g., M3) and 20-week (e.g., M5) time points during the strength-training program. Cardiopulmonary exercise testing was conducted before and after the 20-week strength training program to confirm that the program focused specifically on enhancing muscle strength rather than improving exercise capacity. Testing was not performed at 12 weeks, as these parameters were intended as controlled variables at the program’s conclusion rather than as primary outcomes. A comprehensive description of the overall study design has been previously published [25].

### 2.4. Training Program

The detailed resistance-training program protocol conducted in this study involved three supervised weekly sessions spread over a 20-week period, conducted in a fully equipped university sports center dedicated to strength training. The 20-week program encompassed concentric, eccentric, and isometric phases, which were successively repeated periodically at 2- and 4-week intervals throughout this study. Each session included four exercises, totaling eight different exercises over the program duration (Table 1).

**Figure 1:**
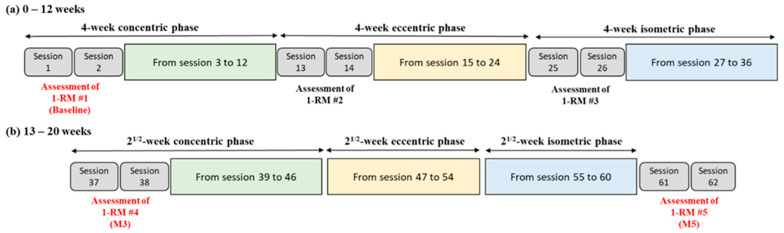
Chronological overview of the 20-week resistance training program with 1-RM assessment.

These exercises targeted both upper and lower body muscle groups, with a rotation scheme implemented across two sessions to ensure each muscle group was adequately trained while allowing for muscle recovery and injury prevention. To tailor the training program to each individual’s progress, we conducted 1-RM assessments every 3 weeks for each exercise task presented in Table 1. The protocol for assessing 1-RM began by selecting an initial weight that aligns with the participant’s perceived strength capacity. From there, resistance was gradually increased across trials until the participant could no longer complete a full repetition with the selected load. Then, 1-RM was defined as the heaviest weight that could be lifted once, while maintaining the correct lifting technique [26]. These evaluations enabled us to adjust the exercise intensity for subsequent cycles, ensuring the maintenance of high-intensity training throughout the program. Loads were defined as percentages of the 1-RM (the concentric phase of a successful one-repetition maximum), and all sessions were supervised by a coach to ensure safety and proper execution, with particular attention to the eccentric training. The resistance training program is presented in Figure 1 (from Pamart et al., 2023) [25].

Each training session commenced with a 10 min warm-up consisting of a 3 min joint mobilization session targeting the neck, shoulders, wrists, hips, knees, and ankles to prepare these areas for exercise, followed by a moderate-to-high intensity Tabata workout. The latter included five exercises: alternating lunges, jumping jacks, burpees, mountain climbers, and squat jumps. Each exercise was performed in 20 s bursts with 10 s of rest in between, structured into a 2 min, 30 s cycle. This cycle was repeated three times, with 30 s of rest between each set, totaling 7 min and 30 s of intense warm-up activity. In the concentric phase, participants performed sets as follows: 8 reps at 70% of 1-RM, 6 reps at 80%, 3 reps at 90%, and 2 reps at 90%. For the eccentric phase, they completed 4 sets of 6 reps at 90% of 1-RM, 3 sets of 4 reps at 100%, followed by 3 sets of 3 reps at 110% and 120%, and 2 reps at 130%, interspersed with 6 reps at 80%, 8 reps at 70%, and 10 explosive reps at 50%. The isometric phase included 5 sets of 8 stato-dynamic reps at 50%, followed by 5 sets of 4 reps at 50% and 1 rep at 80% held for 20 s. Additionally, participants performed 5 sets with 1 rep at 80% for 20 s and 4 explosive reps at 80%, and 5 sets with 2 reps at maximal isometric held for 6 s each and 6 explosive reps at 80%. Participants were advised against engaging in any additional physical training sessions outside of those outlined in the study protocol, including recreational sports.

### 2.5. Procedures

The following procedures were used in this study as detailed in Section 2.3. Additionally, participants underwent electrocardiograms during the initial medical check-up to identify any contraindications to resistance training. Transthoracic echocardiography (TTE) and actimetry recordings were also conducted to evaluate cardiac function and monitor physical activity and sleep, and were further analyzed in a separate article.

#### 2.5.1. Isokinetic Testing

**Figure 2:**
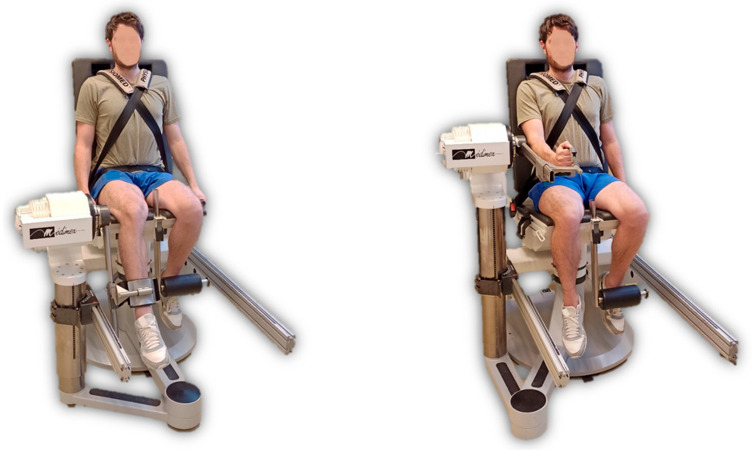
Setup and positioning for knee and elbow flexion/extension testing.

Isokinetic testing was assessed using an isokinetic Con-Trex^®^ isokinetic dynamometer (Con-Trex MJ; CMV AG, Dübendorf, Switzerland). The testing apparatus was set up as described in the manufacturer’s manual and participants were positioned in the seated position for knee flexion/extension testing and elbow flexion/extension testing (Figure 2).

For all tests, the dominant limb was considered the preferred limb for writing or kicking a ball for the upper and lower limbs, respectively. All participants had a 10 min warm-up and familiarization set of submaximal repetitions for all conditions. At all assessments, the limb was passively weighted to provide gravity compensation data, and corrections were incorporated. The studied variables were the maximal peak torque (PT, in Newton meter, Nm) for all muscle groups and velocities.

For knee flexors/extensors, the trials were performed in 15–95° flexion–extension ROMs (0° corresponding to knee fully extended), and the data were collected from 20 to 90° flexion–extension ROMs. Data were collected from a first set of 4 maximal repetitions at 60°·s^−1^ in the concentric mode and then a second set of 4 maximal repetitions at 240°·s^−1^ in the concentric mode with constant verbal stimulation [27]. For the elbow flexors/extensors, the trials were performed in 0–75° flexion–extension ROMs (0° corresponding to the elbow fully extended), and the data were collected from 5 to 70° flexion–extension ROMs. Data were collected from a first set of 4 maximal repetitions at 60°·s^−1^ in the concentric mode and then a second set of 4 maximal repetitions at 150°·s^−1^ in the concentric mode with constant verbal stimulation [28]. Rest periods were 1 min between sets and 5 min between testing different limbs.

#### 2.5.2. Assessment of the F-V Relationship from Isokinetic Measurements

**Figure 3:**
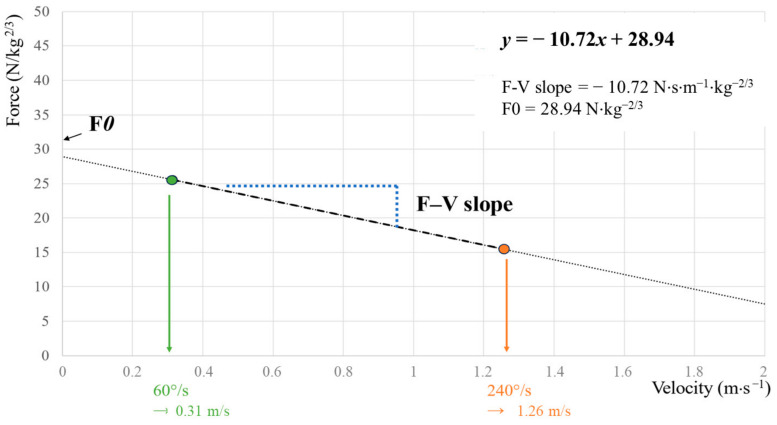
Example of the linear regression models obtained from the force and velocity data during the knee extension using data measured at 60°/s and 240°/s (data were obtained from one participant and assessed at Baseline).

PTs and angular velocities were converted to force and linear velocity by dividing or multiplying, respectively, the value by the radius, which corresponded to the subject’s lever arm length. Force values were normalized to body size (N/kg^2/3^) and body mass was assessed with a mechanical flat scale (Seca 750, Hamburg, Germany). The theoretical maximum force (F0) was calculated by extrapolating the regression line to its limits (i.e., zero velocity) and the slope of the relationship was calculated as the slope of the regression line (F-V slope, in N·s·m^−1^·kg^−2/3^) (Figure 3.) [17,20]. The F-V slope represents the index of the athlete’s individual balance between force and velocity capabilities with a steeper slope corresponding to a more “force-oriented” F–V profile [3].

#### 2.5.3. Cardiopulmonary Exercise Testing

All participants underwent a comprehensive, incremental stepwise exercise test on an electronically braked ergometer (Ergoline, Ergoline GmbH) to assess their cardiopulmonary function. Continuous heart rate (HR) monitoring and 12-lead ECG tracking were implemented throughout the test to ensure real-time cardiovascular observation. The protocol began with a 1 min resting phase in the seated position, followed by a 3 min warm-up period at a load of 50 watts. The workload then increased by 30 watts every 3 min until participants reached volitional exhaustion, supported by verbal encouragement to promote maximum exertion.

To monitor cardiovascular responses, blood pressure was measured every 2 min using a manual cuff. Respiratory gas exchange was analyzed breath-by-breath with the Medisoft gas analyzer (ExpAir Soft), following previously validated methods [29]. The gas analysis system was calibrated before and after each test according to manufacturer specifications to ensure precision. Key parameters, including maximum oxygen uptake ($\dot{V}$O_2peak_) with ventilatory threshold (VT1) were assessed by two trained, independent operators. $\dot{V}$O_2peak_, an indicator of exercise capacity, was determined as the average value recorded during the last 20 s of exercise. Test adequacy was confirmed through a respiratory exchange ratio (RER) greater than 1:1 and an HR at peak exercise exceeding 85% of the estimated maximal HR [30]. All measured values were indexed to body weight to provide standardized, comparable metrics across participants.

#### 2.5.4. Body Composition

Body composition was assessed using bioelectrical impedance analysis (mBCA 525 SECA, Hamburg, Germany) with participants in a supine position. This device employs an 8-electrode configuration, allowing for segmental impedance measurement, and is a validated tool for estimating body composition, including both body fat and skeletal muscle mass [31,32]. Adhesive gel electrodes were positioned at specified anatomical points on the dorsal sides of the hand, wrist, ankle, and foot, following the manufacturer’s guidelines. Each BIA measurement took 75 s to complete. The data collected were total fat mass (both in absolute terms in kg and as a percentage of body mass) as well as skeletal muscle mass (in kg and % of body mass) for subsequent analysis.

### 2.6. Statistics

The data are presented in mean ± standard deviation (SD). Normal distribution was verified using a Shapiro–Wilk test for parametric statistics. The changes in strength parameters were tested with a repeated measure ANOVA with eta squared (η^2^) used for measuring the effect size and classified as small (η^2^ = 0.01), medium (η^2^ = 0.06), or large (η^2^ > 0.14). The difference in F-V parameters between agonistic muscles was tested using a paired *t*-test. A repeated measure correlation (rmcorr) tested the association between the changes in strength parameters measured with the F-V two-point method and the 1-RM procedures. The significance threshold was set at *p* < 0.050 for all tests. The analyses were performed using SPSS for Windows, version 25.0 (SPSS Inc., Chicago, III., USA), and Shinny for rmcorr. Sample size calculation based on Janicijevic et al. showed that this study would require a sample size of 19 participants (power of 80%, level of significance of 5%) for detecting an effect size of 0.72 between pairs [5].

## 3. Results

### 3.1. Characteristics of Participants

A total of 27 individuals participated in this study and 22 participants (81.5%) completed the protocol. Reasons for dropout were lost to follow-up after the 12-week assessment for four participants and one participant was excluded for medical reasons outside the protocol. Among the 22 participants, 6 were excluded from the final analysis because of missing data for at least one measurement at any angular velocity. The characteristics of the 18 participants included (100% male, 22.0 ± 3.4 years) are presented in Table 2.

The participants completed a median of 60 sessions, with all participants having completed the entire program except for two. No significant injury was reported during the protocol. As anticipated, there was no significant improvement in $\dot{V}$O_2peak_.

### 3.2. Changes in F-V Profile

The changes in the F-V profile for the knee and elbow flexor/extensor muscles are reported in Table 3.

There was a significant increase in F0 for all muscle group tests on both the dominant and non-dominant sides (*p* < 0.050), except for elbow flexors. The average increase in F0 was 30–34% for knee muscles and 12–20% for elbow muscles, with effect sizes classified as large (η^2^ > 0.14, 0.262–0.883), except for the dominant-side elbow flexors (η^2^ = 0.131). Post hoc analysis showed a significant increase (*p* < 0.001) between Baseline and M3 for knee flexors and knee extensors, and elbow extensors on the dominant side. There was a significant improvement between M3 and M5, only for the dominant lower limb.

**Figure 4:**
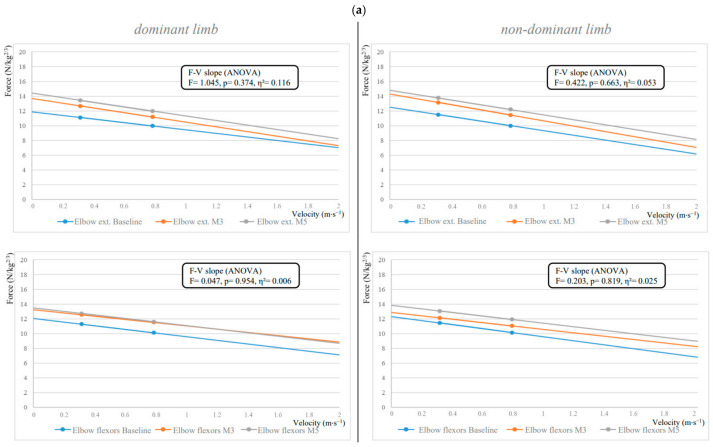

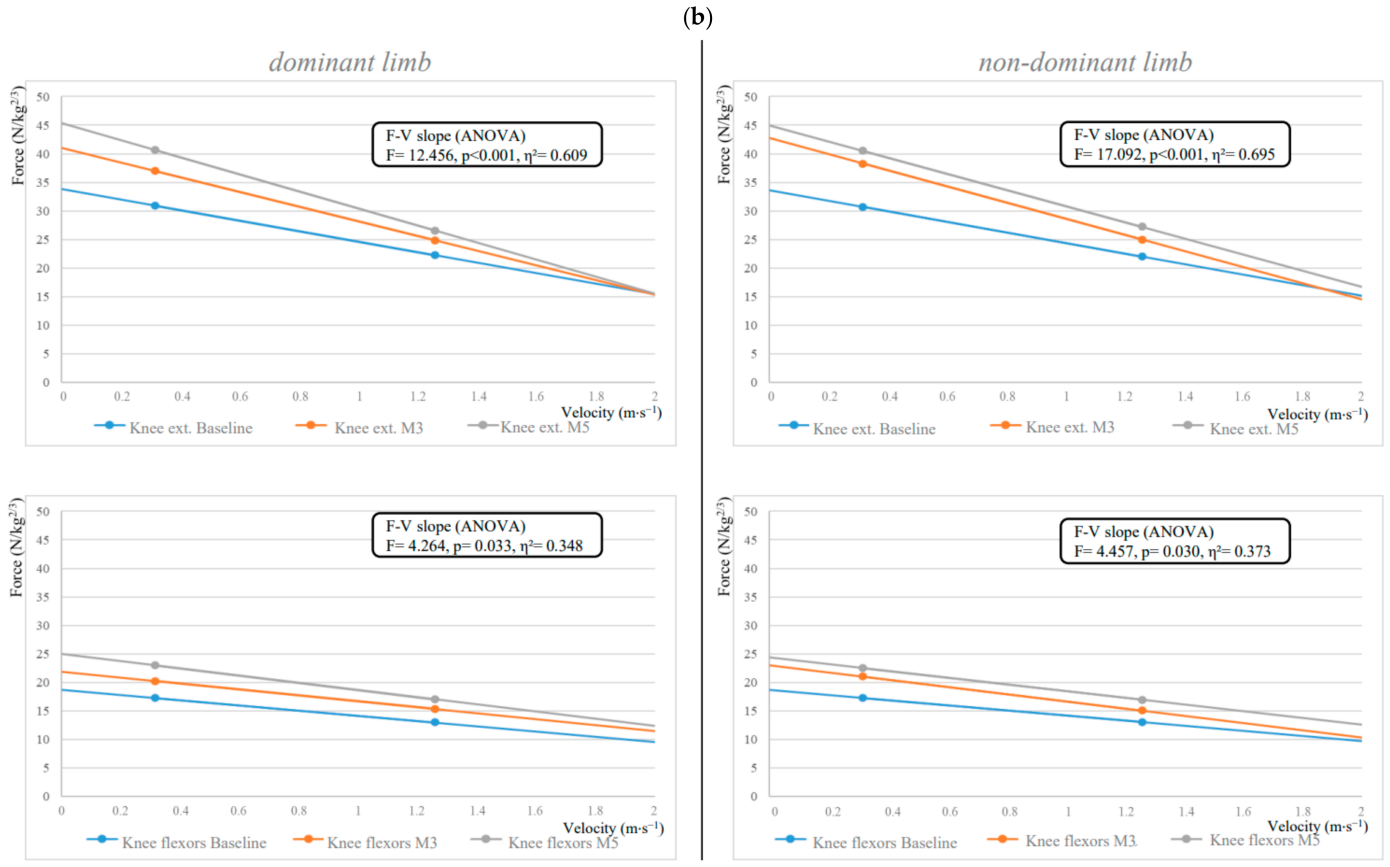
Force–velocity parameter changes illustrated through linear regression models of force and velocity data during (**a**) elbow extension and flexion and (**b**) knee extension and flexion isokinetic tasks with repeated measures ANOVA. ext: extensors.

There was a significant decrease in F-V slope, corresponding to a steeper slope, for knee extensors and flexors on both dominant and non-dominant limbs with effect sizes classified as large (η^2^ > 0.14, 0.348–0.695) (Figure 4). Post hoc analysis showed a significant increase (*p* < 0.001–0.010) between Baseline and M3 or M5 for knee flexors and knee extensors.

#### Differences in Agonistic Muscles

For testing differences in the F-V parameter changes between agonistic muscles, an interaction analysis was performed including the variable time (change over time) and muscle groups (agonist vs. antagonists).

For the upper limbs, there was no significant time × muscle groups interaction effect for the F0 parameter on both the dominant (F = 0.857, *p* = 0.443, η^2^ = 0.097) and non-dominant sides (F = 1.656, *p* = 0.224, η^2^ = 0.181). In addition, there was no significant interaction effect for the F-V slope on both the dominant (F = 1.023, *p* = 0.382, η^2^ = 0.113) and non-dominant sides (F = 0.612, *p* = 0.555, η^2^ = 0.075).

For the lower limbs, there was a significant time × muscle groups interaction effect for the F0 parameter for both the dominant (F = 17.460, *p* < 0.001, η^2^ = 0.686) and non-dominant sides (F = 6.581, *p* = 0.009, η^2^ = 0.467) with a greater increase in F0 for knee extensors compared to flexors. In addition, there was a significant interaction effect for the F-V slope for both the dominant (F = 12.536, *p* < 0.001, η^2^ = 0.610) and non-dominant sides (F = 4.95, *p* = 0.022, η^2^ = 0.398) with a more negative (steeper) F-V slope over time for knee extensors compared to flexors

### 3.3. Associations Between F-V Parameters and 1-RM Procedure

The associations between F-V parameters and the 1-RM procedure, for corresponding muscle groups, are presented in Table 4.

Repeated measures correlations found that the changes in F0 were correlated with the changes in 1-RM for the corresponding muscle groups and for all analyses performed (all *p* < 0.001 except F0*_elbow flexors_* with 1-RM*_arm curl_*, *p* = 0.019). Correlations were strong (r > 0.70) for all exercises performed at the lower extremities.

The changes in F-V slope for knee extensors were negatively associated with the changes in 1-RM in both leg extension and leg press tests (*p* < 0.001) with an almost strong negative correlation (r = −0.62 and −0.67 for leg extension and press, respectively), corresponding to a steeper slope associated with a greater gain in 1-RM for these tests. There was a significant negative association between the flexors’ F-V slope and 1-RM gain on leg curls for the non-dominant limb (*p* = 0.034). No significant association was found between the changes in the upper limb F-V slope and the 1-RM tests. Surprisingly, the correlation between the extensors’ F-V slope and bench press gains significantly differed between the dominant (r = −0.32) and non-dominant limbs (r = −0.01).

## 4. Discussion

The main findings were that the 20-week resistance training program significantly increased F0 and shifted the F-V profile to a more “force-oriented” profile, particularly for knee muscles. Additionally, there were significant differences indicating that knee extensors showed greater improvement in the force domain compared to knee flexors. Finally, the changes in F-V parameters correlated with 1-RM performance, especially for knee muscles.

This 20-week resistance training program induced a more “force-oriented” F-V profile in lower limb muscles, which could be beneficial for maximizing performance in athletes specializing in developing force qualities [7]. The interpretation of the meaningfulness of changes in F0 and F-V slope is limited due to the absence of established minimal clinically important differences (MCIDs) or minimal detectable changes (MDCs). However, effect sizes were large for almost all muscle groups in F0 and for knee muscles regarding the F-V slope. Furthermore, the F0 data collected at 3 and 5 months not only align with but also surpass previous findings in active individuals, underscoring the significance of these results [5]. This substantial and rapid increase in strength (exceeding 30% for the F0 of knee muscles) aligns with established knowledge that resistance exercise studies (conducted over 8 to 12 weeks) typically show a significant initial rise in strength, primarily attributed to neural adaptations [33]. While optimizing the F-V slope linked to an athlete’s F-V profile may enhance athletic and functional performance [9], the extent of change required to achieve a meaningful impact remains unclear. Further research is needed to evaluate the significance of changes in F-V parameters.

Interestingly, Janicijevic et al. tested the sensitivity of the isokinetic method to discriminate between individuals with different physical activity levels and found that the F-V model was highly sensitive for knee muscles but less for elbow muscles [5]. The present results corroborate these findings, highlighting that the F-V model showed satisfactory sensitivity to change to study the adaptations to resistance training in both F0 and F-V slope for knee muscles, but not for elbow muscles. In addition, the correlations between the changes in F-V slope and 1-RM were significant but only for knee muscles. Thus, the use of the two-point isokinetic model for assessing the F-V profile would be particularly sensitive and valid for evaluating the mechanical behavior of knee muscles, but less appropriate when testing the elbow joint. Several hypotheses can be considered. It has been suggested that neural adaptations following resistance training might be limited in the elbow muscles compared to knee extensors, especially when assessing muscle electromyography during maximal voluntary tasks [34]. Also, performing high-velocity movement at the upper limb could be challenging with isokinetic testing and this could be a limit to applying this model, especially for high-velocity domains.

Regarding agonistic muscle function, the results partially confirmed our hypothesis with a significant time × muscle groups interaction effect for the lower limb but not for the upper limb muscles. The findings support that knee extensors had a significantly greater increase in F0 with a steeper F-V slope over time compared to knee flexors. Interestingly, Janicijevic et al. explored whether the two-point isokinetic model for assessing the F-v relationship could discriminate between antagonist muscle groups and demonstrated that F0 and F-v slope were higher for knee extensors compared to knee flexors, but no significant difference for elbow extensors/flexors was found [5]. In addition, the concentric agonistic hamstring-to-quadriceps (H/Q) ratio is correlated with isokinetic velocity resulting in a significantly lower H/Q ratio at the lowest angular velocities compared to the highest velocities, especially in males [35]. The present results corroborate these findings, showing that knee agonistic muscles not only have significant differences for F0 and F-V slope, but the parameters also show significant change over time. Different muscle architecture and muscle physiology between knee extensors and knee flexors may support these findings. The hamstring muscle has a greater proportion of fast-twitch (type II) muscle fibers [36] and could be more “velocity-oriented” compared to the quadriceps. However, if muscle fiber typology could be associated with high-intensity running performance [37], it is uncertain that the muscle composition of hamstring muscles is fully correlated with muscle mechanical properties [38,39]. In addition, the architecture and contractile capacity of the human pennate muscle are inter-related with the larger cross-sectional area (CSA) associated with greater force generation ability and specific adaptation responses evoked by intensive resistance training, especially in knee extensors [40].

There were significant associations between F-V parameters and 1-RM performance across the repeated measurements for each participant. Previous studies have investigated the association between isokinetic and 1-RM performances as well as the changes after a resistance training program [22,41]. However, the present study is the first study to explore the F-V parameters and to demonstrate that, after a resistance training program, repeated measurements of F0 and 1-RM were correlated and exhibited similar patterns. Indeed, Rivière et al. investigated the position of the 1-RM squat point on the F-V relationship obtained during the squat jump and showed that the force developed at the 1-RM was ~11% lower than F0 [23]. Additionally, a steeper F-V slope was also correlated with a greater 1-RM, but only for knee muscles. Thus, not only maximal force but also adaptations to a more “force-oriented” F-V profile over time were associated with greater performances in 1-RM. However, caution is advised when comparing exercises as F-V profiling is task-specific and does not accurately represent the intrinsic force–velocity relationship of the lower extremities [42]. The correlation between F-V parameters and 1-RM performance was similar for both sides, except for the elbow extensors’ F-V slope. Interestingly, Krzysztofik et al. found that muscle activity in the triceps brachii was higher on the dominant side (vs. non-dominant) during a bench press exercise and especially at 90% of 1-RM (compared to 50%) [43]. Therefore, it is likely that the force–velocity pattern of the triceps brachii during a bench press exercise differs between the dominant and non-dominant limbs.

The findings of this study have practical implications for strength and conditioning professionals. The observed increase in F0 and the steeper F-V slope in knee flexors/extensors, but not in elbow muscles, suggest that the resistance training program was effective in developing a “force-oriented” profile in lower limbs. This implies that the two-point isokinetic model is particularly sensitive and valid for assessing adaptations in knee muscles, making it a valuable tool for tracking progress and optimizing training programs focused on lower limb strength. Additionally, the correlation between changes in F-V parameters and 1-RM performance highlights the utility of F-V profiling in predicting and monitoring strength gains. However, the model’s limited sensitivity for elbow muscles suggests the need for alternative assessment methods for upper limbs.

This study has several limitations that should be considered. The small sample size limits the findings, and further studies with larger populations are needed to enable subgroup analyses, particularly including female participants and diverse athletic populations. The use of self-reported data for drug and ergogenic product use could be a limitation, as it may not have been thoroughly verified. Conducting systematic blood tests and monitoring selected biological variables over time would have provided more robust data. Additionally, the lack of randomization and a control group limits our ability to determine whether the observed changes in the F-V profile are solely due to the training intervention, as we cannot compare it to other types of training. Also, isokinetic testing is particularly valuable for assessing force–velocity parameters in novice individuals, as it offers a safe, low-fatigue method designed to measure muscle strength across a broad velocity spectrum. However, the isolated nature of the movement patterns used may lack ecological validity, as they may not fully reflect real-world activities, particularly those involving multi-joint movements. Despite this, our findings show that changes in isokinetic testing correlated with those from non-laboratory assessments, such as 1-RM tasks, supporting its relevance in various settings. Participants received financial compensation, as strongly recommended by the ethics committee, to cover transportation costs and as a token of appreciation for their participation in this study. If any bias was introduced, it would primarily be a selection bias, attracting individuals who might be motivated by the reward. However, the compensation was modest, and response bias was unlikely since the outcomes were based on objective measures rather than subjective assessments. Finally, while the changes in the force–velocity profile are statistically significant, their practical implications for coaches and athletes require further investigation. Future research should explore the effectiveness of the F-V method among more experienced athletes and across diverse populations, such as women and older adults, to address these gaps.

## 5. Conclusions

This 20-week resistance training program significantly increased F0 while shifting the F-V profile to a more “force-oriented” profile, especially for knee muscles, which also exhibited significant agonistic differences. Additionally, these changes correlated with improvements in 1-RM performance, though less so for elbow muscles. Altogether, these results indicate the validity and sensitivity of the isokinetic 2-point model in assessing changes in the F-V profile, especially at the knee joint. Future studies should incorporate a longer follow-up period and a larger sample size to deepen specific knowledge and allow for comparisons across genders, age groups, and varying athletic levels. Additionally, it would be valuable to investigate alternative methodologies to optimize the assessment of the force–velocity profile for upper limb muscles.

## Figures and Tables

**Table 1:** Overview of exercise tasks performed during each session.

	Session 1	Session 2	Session 3
Exercise #1	Leg extension	Inclined press	Leg extension
Exercise #2	Leg curl	Preacher curl	Leg curl
Exercise #3	Lat pull-down	Machine pullover	Lat pull-down
Exercise #4	Bench press	Butterfly	Bench press

**Table 2:** Characteristics of participants (*n* = 18).

Variables	Baseline	M3	M5	F Stat	*p*-Value	Effect Size (η^2^)
Age (years)	22.0 ± 3.44	-	-			
Height (m)	1.78 ± 0.06	-	-			
Weigh (kg)	70.2 ± 12.0	72.3 ± 11.1 *	72.4 ± 12.1 *	10.603	<0.001	0.570
BMI (kg/m^2^)	22.1 ± 3.3	22.7 ± 3.0 *	22.8 ± 3.3 *	9.717	0.002	0.548
Body fat mass (kg)	11.3 ± 7.5	11.3 ± 7.6	12.1 ± 7.5	1.732	0.209	0.178
Skeletal muscle mass (kg)	29.0 ± 3.7	30.4 ± 4.0 *	29.9 ± 3.7 *	24.000	<0.001	0.750
$\dot{V}$O_2peak_ (mLO_2_‧kg^−1^‧min^−1^)	42.2 ± 7.7	-	42.3 ± 6.0	0.002	0.963	0.000
VT1	27.7 ± 8.7	-	27.5 ± 8.0	0.006	0.938	0.000
$\dot{V}$O_2peak_ maximal load (Watts)	185.9 ± 41.2	-	201.8 ± 37.5	18.000	<0.001	0.529

Values are presented as mean ± SD. Abbreviations: BMI: body mass index; $\dot{V}$O_2peak_: maximum oxygen consumption; and VT: ventilatory threshold. * *p* ≤ 0.010 from Baseline measurement (post hoc analysis).

**Table 3:** Changes in force–velocity parameters including (**a**) theoretical maximum force and (**b**) force–velocity slope for knee and elbow flexors/extensors, with time effect analysis (*n* = 18).

**(a) Theoretical Maximum Force** **(F0)**								
			**F0**			**Time Effect**		
			**Baseline**	**M3**	**M5**	**F Stat**	***p*-Value**	**Effect Size (η^2^)**
Elbow flexion		Dominant	12.05 ± 3.15	13.24 ± 3.42	13.49 ± 2.8	1204	0.326	0.131
		Non-dominant	12.24 ± 2.48	12.87 ± 3.51	13.82 ± 3.52	2.659	0.103	0.262
Elbow extension		Dominant	11.87 ± 2.33	13.69 ± 2.70 ^†††^	14.40 ± 2.43 ^†††^	11.994	<0.001	0.600
		Non-dominant	12.41 ± 3.06	14.27 ± 3.11	14.84 ± 2.95	5.501	0.016	0.423
Knee flexion		Dominant	18.72 ± 4.40	21.90 ± 3.95 ^†††^	24.97 ± 5.52 ^†††,^***	20.323	<0.001	0.718
		Non-dominant	18.65 ± 4.59	23.04 ± 4.56 ^†††^	24.32 ± 5.81	14.659	<0.001	0.662
Knee extension		Dominant	33.81 ± 6.96	41.02 ± 5.76 ^†††^	45.32 ± 8.08 ^†††,^**	60.101	<0.001	0.883
		Non-dominant	34.19 ± 8.48	43.37 ± 8.00 ^†††^	45.57 ± 8.62 ^†††^	35.820	<0.001	0.827
**(b) Force–Velocity Slope** **(F-V slope)**								
			**F-V Slope**			**Time Effect**		
			**Baseline**	**M3**	**M5**	**F Stat**	***p*-Value**	**Effect Size (η^2^)**
Elbow flexion	Dominant		−2.51 ± 1.86	−2.32 ± 2.06	−2.37 ± 1.85	0.047	0.954	0.006
	Non-dominant		−2.47 ± 2.31	−2.19 ± 2.01	−2.40 ± 2.14	0.203	0.819	0.025
Elbow extension	Dominant		−2.42 ± 2.14	−3.20 ± 1.92	−3.09 ± 2.32	1.045	0.374	0.116
	Non-dominant		−2.89 ± 2.01	−3.50 ± 2.11	−3.41 ± 2.34	0.422	0.663	0.053
Knee flexion	Dominant		−4.58 ± 3.43	−5.21 ± 2.49	−6.31 ± 2.67 ^†,^*	4.264	0.033	0.348
	Non-dominant		−4.47 ± 2.49	−6.35 ± 3.06 ^†^	−5.86 ± 2.24 ^†^	4.457	0.030	0.373
Knee extension	Dominant		−9.20 ± 4.67	−12.86 ± 3.73 ^††^	−14.92 ± 4.56 ^†††^	12.456	<0.001	0.609
	Non-dominant		−9.71 ± 5.00	−14.41 ± 4.27 ^†††^	−14.36 ± 3.93 ^†††^	17.092	<0.001	0.695

^†^: significant difference from Baseline (^†^: *p* ≤ 0.050, ^††^: *p* ≤ 0.010, and ^†††^: *p* ≤ 0.001); *: significant difference between M3 and M5 (*: *p* ≤ 0.050, **: *p* ≤ 0.010, and ***: *p* ≤ 0.001).

**Table 4:** Association between changes in isokinetic force–velocity parameters and 1-RM performance: a repeated measures correlation analysis (*n* = 18).

			Dominant		Non-Dominant	
Isokinetic Testing	1-RM	F-V Parameter	r [95% CI]	*p*-Value	r [95% CI]	*p*-Value
Knee extensors	Leg extension	F0	0.83 [0.693, 0.913]	0.001	0.83 [0.682, 0.912]	0.001
		F-V slope	−0.69 [−0.829, −0.456]	0.001	−0.62 [−0.791, −0.355]	0.001
Knee extensors	Leg Press	F0	0.82 [0.665, 0.904]	0.001	0.83 [0.682, 0.912]	0.001
		F-V slope	−0.68 [−0.827, −0.45]	0.001	−0.67 [−0.82, −0.423]	0.001
Knee flexion	Leg curl	F0	0.72 [0.51, 0.85]	0.001	0.68 [0.443, 0.827]	0.001
		F-V slope	−0.29 [−0.566, 0.051]	0.094	−0.37 [−0.626, −0.031]	0.034
Elbow flexion	Preacher curl	F0	0.38 [0.056, 0.634]	0.024	0.38 [0.049, 0.637]	0.026
		F-V slope	−0.07 [−0.394, 0.269]	0.688	0.09 [−0.259, 0.413]	0.627
Elbow extension	Bench press	F0	0.63 [0.37, 0.794]	0.001	0.51 [0.209, 0.724]	0.002
		F-V slope	−0.32 [−0.594, 0.01]	0.057	−0.01 [−0.349, 0.327]	0.943

## Data Availability

The data presented in this study are available upon request from the corresponding author due to privacy.
